# Strong antitumor efficacy of a pancreatic tumor‐targeting oncolytic adenovirus for neuroendocrine tumors

**DOI:** 10.1002/cam4.1185

**Published:** 2017-09-21

**Authors:** Yuki Yamamoto, Masaki Nagasato, Yosei Rin, Marina Henmi, Yoshinori Ino, Shinichi Yachida, Rieko Ohki, Nobuyoshi Hiraoka, Masatoshi Tagawa, Kazunori Aoki

**Affiliations:** ^1^ Division of Molecular and Cellular Medicine National Cancer Center Research Institute Tokyo Japan; ^2^ NCC Cancer Science Tokyo Medical and Dental University Tokyo Japan; ^3^ Molecular Pathology National Cancer Center Research Institute Tokyo Japan; ^4^ Cancer Genomics National Cancer Center Research Institute Tokyo Japan; ^5^ Rare Cancer Research National Cancer Center Research Institute Tokyo Japan; ^6^ Division of Pathology and Cell Therapy Chiba Cancer Center Research Institute Chiba Japan

**Keywords:** Neuroendocrine tumor, oncolytic adenovirus, pancreatic tumor, survivin promoter, targeting ligand

## Abstract

Although oncolytic adenoviruses are promising cancer therapy agents, for effective oncolytic activity, viruses need to specifically infect and effectively replicate in cancer cells but not in normal cells. We have previously identified a pancreatic cancer‐targeting ligand, SYENFSA (SYE), by screening an adenovirus library displaying random peptides against human pancreatic cancer cells and reported that a survivin promoter‐regulated adenovirus, displaying the SYE ligand (AdSur‐SYE), provided effective oncolysis of pancreatic ductal adenocarcinoma (PDAC) in a preclinical study. As we examined the infectivity of AdSur‐SYE in human surgical specimens of various pancreatic tumors, we unexpectedly found that AdSur‐SYE showed high gene transduction efficiency for pancreatic neuroendocrine tumors (PNETs) as well as for PDAC, 9.1‐ and 6.2‐fold, respectively, compared to that of the nontargeting virus (AdSur). The infectivity of both vectors was almost the same in other cancers and organs such as the pancreas. Immunostaining indicated that the cells infected with AdSur‐SYE were PNET cells but not stromal cells. AdSur‐SYE showed a significantly higher oncolytic potency than that of AdSur in human PNET cell lines, and intratumoral infection with AdSur‐SYE completely diminished subcutaneous tumors in a murine model, in which AdSur‐SYE effectively proliferated and spread. AdSur‐SYE exerted a stronger oncolytic effect in primary PNET cells cocultured with mouse embryonic fibroblasts than AdSur did. Thus, AdSur‐SYE shows promise as a next‐generation therapy for PNET.

## Introduction

Oncolytic viruses, which are capable of specifically replicating in cancer cells, have shown promising results in various cancer models. Oncolysis is initiated by transduction of a small number of cells with a virus, and then the virus replicates and spreads to appropriate cells within the tumor, finally leading to the disappearance of the tumor mass 1, 2, 3. Oncolytic adenoviruses based on human serotype 5 are one of the best‐studied viruses, owing to their high‐titer production and highly effective infection of a wide spectrum of dividing and nondividing cells both in vitro and in vivo. Currently, two types of the adenovirus are used to restrict the viral replication to cancer cells, without affecting normal cells 4, 5. The first type introduces a mutation in the adenoviral E1A gene; the mutated gene is functionally complemented by genetic mutations in cancer cells, such as p53 mutations or abnormalities in the retinoblastoma pathway. The second type involves the construction of adenoviruses in which the transcription of E1 genes is restricted to cancer cells by tumor‐ or tissue‐specific promoters such as the prostate‐specific antigen, survivin, midkine, and telomerase reverse transcriptase promoters 4, 5. Oncolytic adenoviruses have been tested in patients with malignancies, without showing side effects, and several clinical trials are ongoing 1, 4, 6.

Progress in clinical studies of oncolytic adenoviruses has indicated that a cancer‐targeting strategy is necessary to enhance the antitumor efficacy and to ensure the safety of patients 7. Several strategies have been developed to redirect the tropism of the adenovirus vector to permit efficient target gene delivery to specific cell types. One approach is to link a ligand for cell surface receptors with capsid proteins of the adenovirus vector via bispecific conjugates. However, this approach is not useful for oncolytic virotherapy, since the adaptor‐vector complex is not formed during virus replication in the tumors. In another approach, chimeric adenoviruses are created usually based on serotype 5 with the fiber or its knob domain replaced by that of another serotype. This approach is also hampered by the restriction of the replaced serotype tropism. Therefore, retargeting of adenovirus vectors by the incorporation of ligands directly into the fiber proteins is preferable 7.

This type of targeting can create a tissue tropism to new cellular targets by conferring novel, ligand‐mediated receptor‐binding properties to viruses and simultaneously inhibiting their binding to natural receptors (detargeting) 7, 8. We have recently constructed an adenovirus library displaying random peptides on a fiber knob to generate adenoviral vectors targeting specific cell types 9, 10, 11. As a result, we have demonstrated that a pancreatic cancer‐targeting sequence, SYENFSA (SYE), which was selected from the adenovirus library by screening against the AsPC‐1 pancreatic cancer cell line, significantly enhanced the gene transduction efficiency of the adenoviral vector in pancreatic cancer cell lines but not in normal cells 12, 13. Subsequently, the SYE sequence was combined with a survivin promoter‐regulated oncolytic adenovirus (AdSur‐SYE), in which the native tropism was ablated 14. AdSur‐SYE showed a higher infectivity and more effective cytotoxicity than the nontargeting oncolytic virus (AdSur) for human pancreatic ductal adenocarcinoma (PDAC) 14.

By comparing AdSur‐SYE and AdSur using surgical specimens of various pancreatic tumors, we unexpectedly found that the transduction efficiency of AdSur‐SYE was dramatically higher in pancreatic neuroendocrine tumors (PNET) than that of AdSur. PNETs are rare tumors, accounting for only 1–2% of all pancreatic neoplasms 15. However, the incidence of PNET has increased two‐ to threefold in the last two decades 16, 17. Histologically, PNET originate from hormone‐producing islet cells and are characterized by clinical behaviors ranging from indolent to highly malignant. The pathogenesis of PNET is largely unknown, but approximately 10% of PNET are components of genetic syndromes such as multiple endocrine neoplasm type I, von Hippel–Lindau, and tuberous sclerosis syndromes.^16, 17^ Traditional medical treatment of advanced PNET includes anticancer agent‐based or streptozotocin‐based chemotherapy. Recently, targeting therapeutic agents, especially those that inhibit molecules involved in angiogenesis or growth factor receptor‐related signaling pathways, have revolutionized the treatment strategy 15, 16, 17. However, clinical effects of these therapies are still limited, and the reported 5‐year survival rate is 30% for a nonfunctioning metastatic tumor 16, 17. Thus, novel approaches to improve patient survival are eagerly awaited 15, 16, 17.

Here, we demonstrated in a preclinical study that AdSur‐SYE showed a much higher infectivity and oncolytic potency for PNET as well as for PDAC compared to AdSur. This study provides the rationale for the use of cancer‐targeting oncolytic adenoviruses in the treatment of PNETs.

## Materials and methods

### Cell lines

We used in this study a human PNET cell line (QGP‐1), human pancreatic neuroendocrine carcinoma cell line (A99), human pancreatic cancer cell line (AsPC‐1), human prostate cancer cell line (PC3), and mouse embryonic fibroblasts (MEFs). AsPC‐1 and PC3 cell lines were obtained from ATCC (Rockville, MD, USA). The QGP‐1 cell line was obtained from the Japanese Collection of Research Bioresources Cell Bank (Osaka, Japan). The A99 cell line was established and cultured as previously reported 18. MEFs were purchased from ReproCell (Kanagawa, Japan) and cultured in DMEM (Wako Pure Chemical Industries, Ltd., Osaka, Japan) with 10% FBS. QGP‐1, AsPC‐1, and PC3 cell lines were cultured in RPMI‐1640 medium (Nissui Pharmaceutical, Tokyo, Japan) with 10% FBS.

### Human surgical specimens

Human surgical specimens [10 PNET, eight PDAC, three intraductal papillary mucinous neoplasm (IPMN), one metastasis of renal cancer, one gallbladder cancer, one duodenal cancer, six pancreas specimens, and one liver specimen] were obtained in accordance with the Declaration of Helsinki Principles and the guidelines of the Ethics Committee of the National Cancer Center (Tokyo, Japan). To examine the infectivity of each virus, for a flow cytometric analysis, pancreatic tumor tissues were mechanically processed into single cells with a sterile scalpel or scissors, and filtered with 100 *μ*m nylon mesh (Cell Strainer; Thermo Fisher Scientific, Waltham, MA). For the preparation of single cells, fibroblasts were not removed, since major population of cells prior to the analysis was tumor cells, not stromal cells. For immunohistochemical staining, tissues were sliced into small pieces (1–4 mm in diameter) with a sterile scalpel. The prepared cells and tissue slices were cultured in RPMI‐1640 medium with 10% FBS.

### Plasmids and recombinant adenovirus vectors

A 0.5‐kb survivin regulatory region 19 was inserted into the pGL3 basic vector (Promega, Madison, WI), which contains the firefly luciferase gene, and the plasmid was designated as pSur‐Luc. The pRL‐SV40 plasmid (Promega) expresses the *Renilla* luciferase gene under control of the SV40 promoter.

Adenoviral vectors were constructed as described previously 10, 12, 19. Ad‐EGFP and AdΔE1‐AP have a wild‐type (WT) fiber, while AdSur‐SYE and AdSur include four point mutations in the AB‐loop of the fiber knob, which reduces coxsackievirus and adenovirus receptor (CAR) binding. AdSur‐SYE includes a SYE sequence in the HI loop on the fiber knob 14. AdSur‐SYE and AdSur contain a 0.5‐kb survivin regulatory region upstream of the adenoviral E1 gene, and Ad‐EGFP contains a WT E1 region. AdSur‐SYE, AdSur, and Ad‐EGFP contain a Cytomegalovirus immediate early (CMV) promoter, the enhanced green fluorescence protein (EGFP) gene, and an SV40 poly(A) signal in place of the E3 region. In AdΔE1‐AP, the E1 gene was replaced with the CMV promoter‐driven alkaline phosphatase (AP) gene. The physical particle concentration [viral particles (vp)/mL] of virus preparations was determined by optical density at 260 nm (OD_260_).

### Luciferase activity assay

Cells were seeded at 1 × 10^4^ cells per well in 96‐well plates. QGP‐1, AsPC‐1, and PC3 cells were transfected with pSur‐Luc or pRL‐SV40 by a lipofection method (X‐tremeGENE HP DNA transfection reagent; Sigma–Aldrich., St. Louis, MO). A99 cells were transfected with plasmids by an electroporation method (Neon electroporation transfection system; Thermo Fisher Scientific). After 48 h, light units of the firefly and *Renilla* luciferase activities were measured by a dual luciferase reporter assay (Promega) using a luminometer (MiniLumat LB9506; EG&G Gerthold, Vilvoorde, Belgium). The relative luciferase activity was calculated as a ratio of light units of cells transfected with pSur‐Luc to those of cells transfected with pRL‐SV40.

### In vitro cell viability assay

Cells were seeded at 3 × 10^3^ cells per well in 96‐well plates and infected with viruses at 100, 3 × 10^2^, 1 × 10^3^, and 3 × 10^3^ vp/cell. Single cells (1 × 10^4^) prepared from surgical specimens of PNETs were cocultured with the same number of MEFs in 96‐well plates and infected with viruses at 300, 1 × 10^3^, 3 × 10^3^, 1 × 10^4^, and 3 × 10^4^ vp/cell. The numbers of viable cells were measured using a premix WST‐1 cell proliferation assay system (Takara Bio, Shiga, Japan).

### In vivo tumor growth

Five‐week‐old female BALB/c nude mice were purchased from Charles River Japan, Inc. (Kanagawa, Japan) and housed under sterile conditions. Animal studies were carried out in accordance with the Guideline for Animal Experiments of the National Cancer Center Research Institute and approved by the Institutional Committee for Ethics in Animal Experimentation. Tumor‐bearing mice were prepared by subcutaneous injection with 5 × 10^6^ cells of QGP‐1. When the tumor mass (~6 mm in diameter) was established, the tumor was directly injected with 0.1 or 2 × 10^10^ vp of a virus in a total volume of 50 *μ*L. The AdΔE1‐AP (2 × 10^10^ vp) did not suppress QGP‐1 tumor growth as compared to PBS did (data not shown), and was used as a control. The short (*r*) and long (*l*) diameters of the tumors were measured, and the volume of each tumor was calculated as (*r* ^2^ × *l*)/2.

### Detection of adenovirus DNA from cells and tumors

Total DNA was extracted from culture cells 1, 3, and 5 days after adenoviral infection, and tumors 2 and 6 days after the intratumoral injection of an adenovirus solution (2 × 10^10^ vp) using a NucleoSpin tissue kit (Macherey–Nagel, GmbH & Co., Duren, Germany). Adenoviral DNA was measured by SYBR Green real‐time PCR using an Eco™ real‐time PCR system (Illumina, Inc., San Diego, CA). Briefly, 50 ng of extracted DNA was added to a final volume of 10 *μ*L per reaction containing 1 × SYBR Green PCR master mix (Applied Biosystems Japan, Tokyo, Japan) and 100 nmol/L primers, E4 upstream (5′‐GGAGTGCGCCGAGACAAC‐3′) and downstream (5′‐ACTACGTCCGGCGTTCCAT‐3′), which amplify a 68‐bp region. For the standard curve to quantify the E4 copy numbers, E4 template DNA with a known copy number (2.4 × 10 − 2.4 × 10^6^) was also analyzed. The E4 thermal cycling conditions were as follows: initial denaturation at 95°C for 10 min, followed by 40 cycles at 95°C for 10 s and 60°C for 30 s.

### Flow cytometry

Flow cytometry was performed to assess the percentage of EGFP^+^ cells. Cells were harvested 24 h after viral infection and stained with a LIVE/DEAD cell viability assay kit (Life Technologies Corp., Carlsbad, CA) for the detection of live cells, followed by fixation with 4% paraformaldehyde (Wako Pure Chemical Industries, Ltd.) for 60 min at room temperature. To detect the expression of Chromogranin A (CgA), one of the markers of neuroendocrine tumors, the cells were stained with monoclonal rabbit anti‐human CgA antibody (EP1030Y; Abcam, Cambridge, UK) followed by staining with Alexa Fluor 594‐goat anti‐rabbit IgG (Thermo Fisher Scientific), and analyzed by a LSRFortessa cell analyzer (BD Biosciences, San Jose, CA).

### Immunohistochemistry

The surgical specimens of human pancreatic tumor were processed into sliced pieces (1~4 mm in diameter), and the virus solution was added in the culture medium containing small pieces of PNET tissues. Twenty‐four hours later, the tissues were fixed in 4% paraformaldehyde at room temperature overnight. Cryostat tissue sections (4 *μ*m) were mounted on glass slides. The section was subjected to immunohistochemistry to detect CgA, with a rabbit polyclonal *α*‐human CgA antibody (Dako, Glostrup, Denmark). All the sections were counterstained with DAPI (Life Technologies Corp.). After taking fluorescence microscopy photograph, the section was stained with H&E.

### Statistical analysis

Comparative analysis of the data was performed by the Student's *t*‐test using the SPSS statistical software (SPSS Japan, Inc., Tokyo, Japan). Differences were considered statistically significant at a *P*‐value of < 0.05.

## Results

### High gene transduction efficiency of AdSur‐SYE in surgical specimens of PNETs

**Figure 1:**
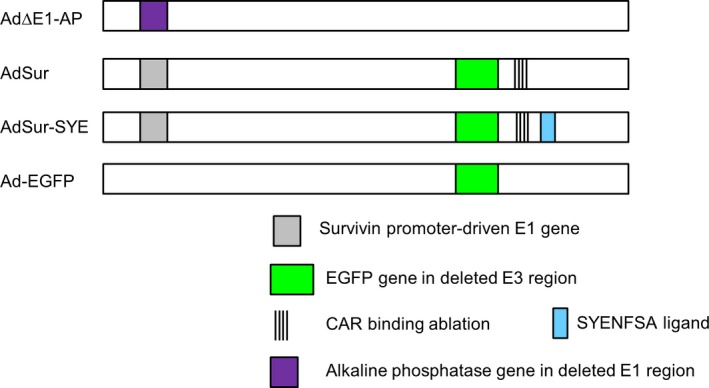
Design of adenovirus constructs displaying a pancreatic tumor‐targeting ligand. In AdSur and AdSur‐SYE, four point mutations were inserted in the AB‐loop to reduce the CAR binding, and the E1 gene is regulated by the survivin promoter. In AdΔE1‐AP, the E1 gene is replaced with the CMV promoter‐driven AP gene. AdSur‐SYE displays a pancreatic tumor‐targeting sequence, SYENFSA, on the fiber knob. Ad‐EGFP has a wild‐type (WT) E1 region and a WT fiber.

**Figure 2:**
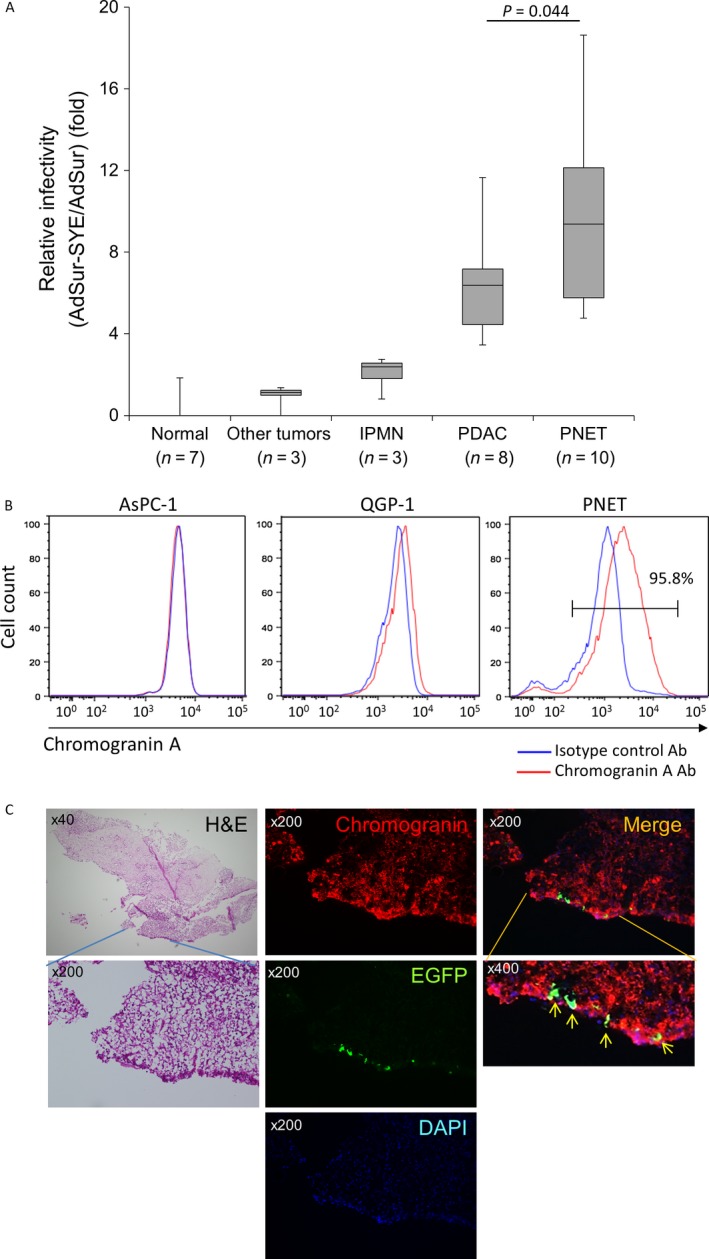
Infectivity of AdSur‐SYE for human PNET tissues. Tumor cells were prepared from surgical specimens of human pancreatic tumors. (A) Detection of EGFP ^+^ cells by flow cytometry. Tumor cells were infected with AdSur or AdSur‐SYE at 1 × 10^3^ vp/cell, and 24 h later, EGFP ^+^ cells were analyzed by flow cytometry. The relative frequency of EGFP ^+^ cells (percentage of EGFP ^+^ cells infected with AdSur‐SYE to that of EGFP ^+^ cells infected with AdSur) is presented. Normal tissues; pancreas (*n* = 6) and liver (*n* = 1), other tumors; metastasis of renal cancer (*n* = 1), gallbladder cancer (*n* = 1) and duodenal cancer (*n* = 1). (B) Expression of CgA in PNET cells. The single cells derived from PNET surgical specimens were stained with an *α*‐CgA antibody, and analyzed by flow cytometry. (C) CgA staining of sliced tissues infected with AdSur‐SYE. The sliced tissues (Pt#62) were infected with 1 × 10^10^ vp of AdSur‐SYE, and after fixation, frozen sections were stained with an *α*‐CgA antibody. Arrows, EGFP ^+^CgA^+^ cells.

We have previously reported that AdSur‐SYE showed a higher gene transduction efficiency for PDAC than AdSur. Both vectors have survivin promoter‐regulated E1 region, and the pancreatic tumor‐targeting ligand, SYE, is incorporated in the fiber of AdSur‐SYE (Fig. 1) 14. To examine the infectivity of AdSur‐SYE for various types of pancreatic tumors, single cells prepared from surgical specimens of the patients with IPMN, PDAC, or PNET were infected with AdSur‐SYE or AdSur. Twenty‐four hours after the infection, the percentage of EGFP^+^ cells was analyzed by flow cytometry. AdSur‐SYE showed 2.1‐, 6.2‐, and 9.1‐fold higher gene transduction efficiencies than AdSur for IPMN, PDAC, and PNET, respectively (Fig. 2A). The efficiencies of AdSur‐SYE and AdSur were almost the same in other types of cancer and organs such as the pancreas and liver, as previously reported 14. The patient information of surgical specimens was listed in Table S1. These results showed that the targeting sequence SYE enhanced the gene transduction efficiency of the adenovirus vector not only for PDAC but also for PNET. The flow cytometric analysis showed that major population of single cells prepared from PNET specimens prior to the analysis was positive for CgA, a marker of neuroendocrine tumor cells (Fig. 2B). Flow cytometry also showed that a significant higher frequency of EGFP^+^ cells among CgA^+^ cells in AdSur‐SYE‐infected cells as compared with that in AdSur‐infected cells (Fig. S1).

Next, sliced tissues of PNET surgical specimens were infected with viruses. EGFP^+^ cells were detected on the surface of the AdSur‐SYE‐infected tissues but not in the AdSur‐infected tissues. The EGFP^+^ cells in the AdSur‐SYE‐infected tissue seemed to be the PNET cells that were stained with H&E in the same section (Fig. 2C). Immunostaining showed that most of the EGFP^+^ cells in the AdSur‐SYE‐infected tissues were positive for CgA, indicating that the majority of AdSur‐SYE‐infected cells were PNET cells but not stromal cells such as endothelial and immune cells in the tissue (Fig. 2C).

### High gene transduction efficiency of AdSur‐SYE in PNET cell lines

**Figure 3:**
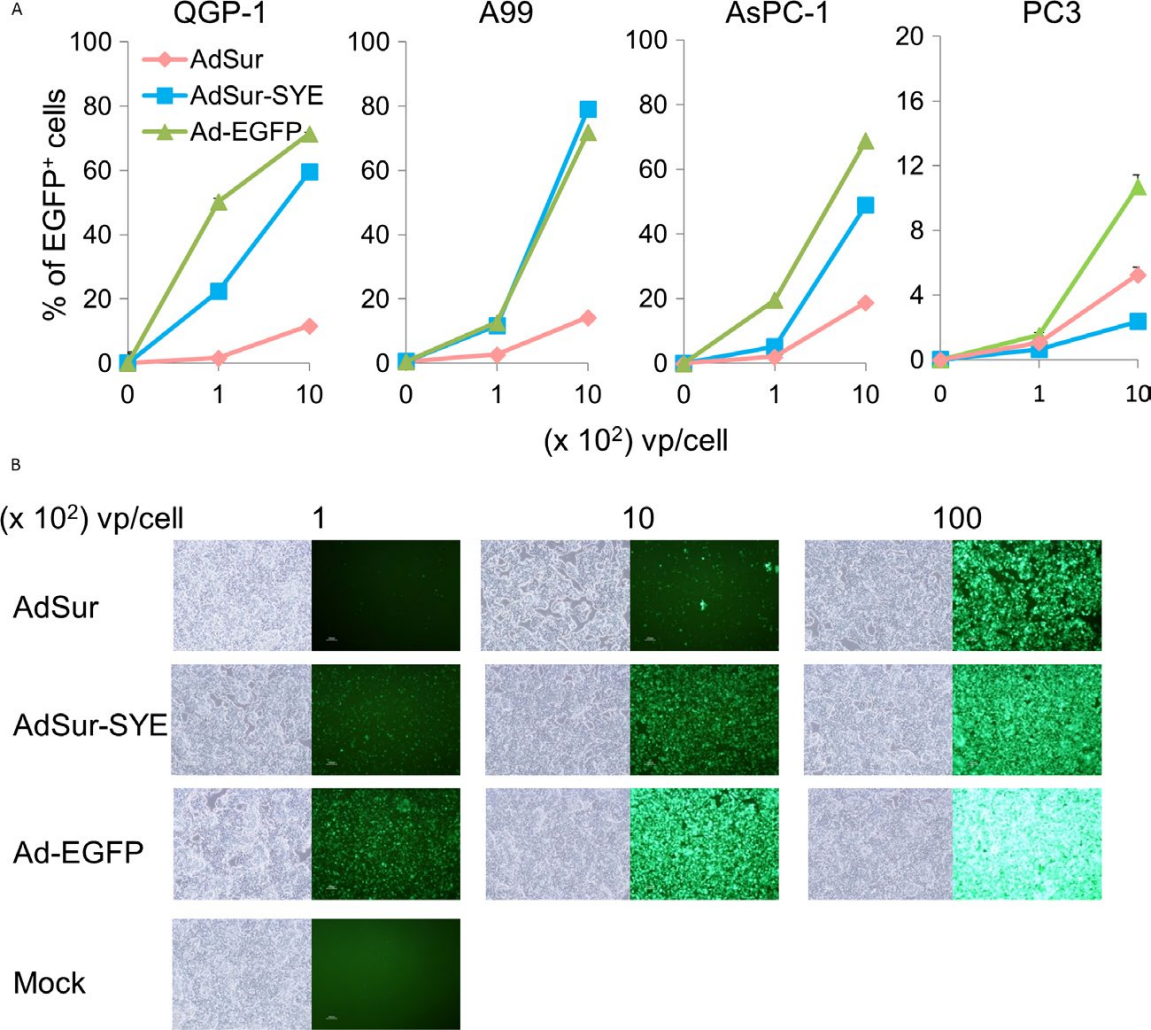
Infectivity of AdSur‐SYE in PNET cell lines. Cells were infected with various viruses and analyzed 24 h later. (A) Percentage of EGFP ^+^ cells. Cells were infected with viruses at 100 or 1 × 10^3^ vp/cell, and EGFP ^+^ cells were analyzed by flow cytometry (*n* = 4). (B) Photographs of QGP‐1 cells. The cells were infected with viruses at 100, 1 × 10^3^ and 1 × 10^4^ vp/cell.

To confirm that AdSur‐SYE had a higher gene transduction efficiency for human PNET cell lines, QGP‐1 and A99, as well as other cell lines, were infected with AdSur, AdSur‐SYE, or Ad‐EGFP. The flow cytometric analysis showed that AdSur‐SYE significantly increased the frequencies of EGFP^+^ cells in both QGP‐1 and A99 cell populations, as well as in that of AsPC‐1 cells, in a dose‐dependent manner, whereas the frequency of EGFP^+^ cells among AdSur‐SYE‐infected cells was rather lower to that among AdSur‐infected PC3 cells (Fig. 3A and B).

### Enhanced activity of the survivin promoter in PNET cells

**Figure 4:**
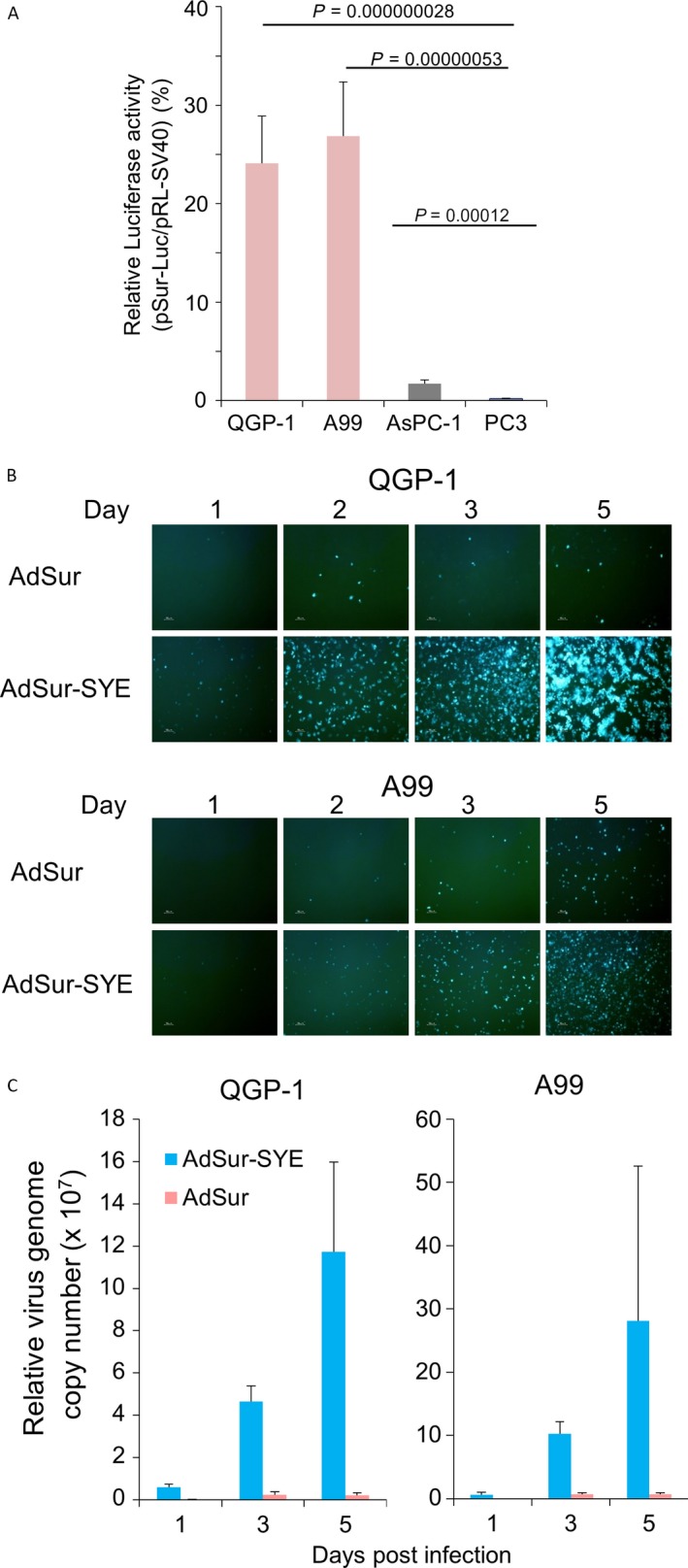
Activity of the survivin promoter in PNET cell lines. (A) Activity of the survivin promoter in human pancreatic cancer and PNET cell lines. Cells were transfected with pSur‐Luc and pRL‐SV40 using lipofection (QGP‐1, AsPC‐1, and PC3) or electroporation (A99), and 48 h later, the luciferase activity was measured. The relative luciferase activity (light units of cells transfected with pSur‐Luc to those of cells transfected with pRL‐SV40) is presented. The assays (*n* = 5) were repeated three times, and the mean ± SD was plotted. (B) Photographs of cell lines. QGP‐1 and A99 cells were infected with the viruses at 1 × 10^3^ vp/cell, and observed by fluorescent microscopy on the indicated days. (C) Replication of adenoviral DNA in cells. QGP‐1 and A99 cells were infected with AdSur‐SYE or AdSur at 1 × 10^3^ vp/cell, and 1, 3, and 5 days later, adenoviral DNA was analyzed by real‐time PCR (*n* = 3). The copy numbers of the indicated viruses and on indicated days are shown with respect to that of AdSur on day 3.

The replication of AdSur and AdSur‐SYE is regulated by the survivin promoter, which has been reported to exhibit a higher activity only in a variety of cancers but not in normal tissues 19, 20. To confirm the relevance of the survivin promoter to the PNET cell lines, cells were transfected with the pSur‐Luc and pRL‐SV40 plasmids. The relative activity of the survivin promoter versus the SV40 promoter was higher in AsPC‐1 cells than in PC3 cells as previously reported 14, while the activity was much higher in QGP‐1 and A99 cells (Fig. 4A).

To examine the viral replication in PNET cell lines, cells were infected with viruses at MOI of 1 × 10^3^ vp/cell and harvested on 1, 3, and 5 days after the infection. A rapid expansion of EGFP^+^ cells was detected in AdSur‐SYE‐infected cells compared to those infected with AdSur (Fig. 4B). Furthermore, real‐time PCR showed that the copy number of the adenoviral genome per cell was more expanded in AdSur‐SYE‐ but not in AdSur‐infected cells (Fig. 4C), suggesting that AdSur‐SYE proceeded faster throughout the cell layer due to improved initial infection and reinfection processes than did AdSur. These data also indicated efficient survivin promoter‐mediated viral replication in PNET cells.

### Cytotoxic effect of AdSur‐SYE

**Figure 5:**
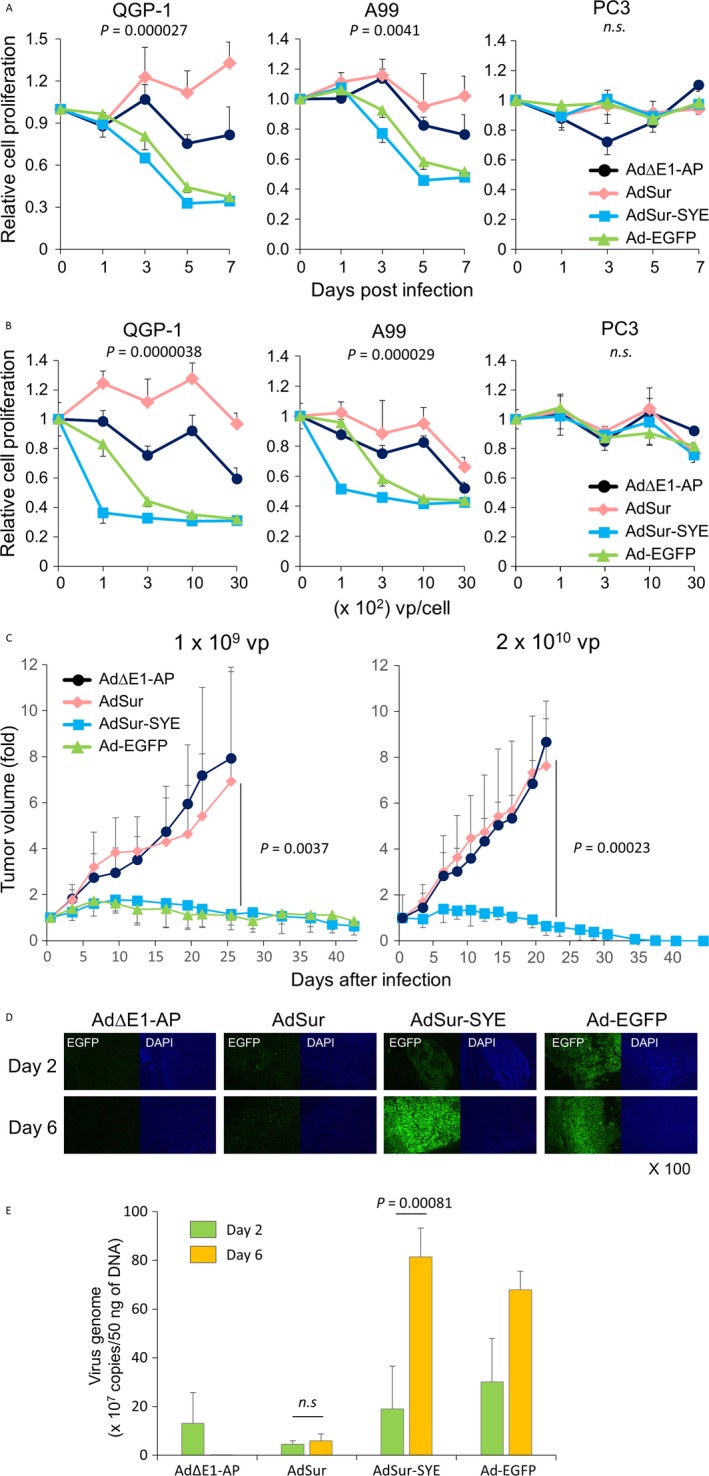
Cytotoxic activity of AdSur‐SYE for PNET cells. (A) Time course of cell growth suppression of PNET cell lines. Cells were infected with viruses at 3 × 10^2^ vp/cell, and 1, 3, 5, and 7 days later, a cell viability assay was performed. The relative cell proliferation (OD_450_ on the indicated day to that on day 0) is shown. The assays (*n* = 4) were repeated two times, and the mean ± SD was plotted. The *P*‐value: AdSur‐infected cells versus AdSur‐SYE‐infected cells on day 5. (B) Suppression of cell proliferation of PNET cell lines at various MOIs. Cells were infected with various amounts of viruses, and 5 days later, a cell viability assay was performed. The relative cell proliferation (OD_450_ on the indicated vp/cell to that at 0 vp/cell) is shown. The *P*‐value: AdSur‐infected cells versus AdSur‐SYE‐infected cells at 1 × 10^3^ vp/cell. (C) Growth suppression of QGP‐1 subcutaneous tumors. Adenovirus vectors were injected into QGP‐1 subcutaneous tumors developed after QGP‐1 cell injection to immune‐incompetent mice. Left, adenoviruses were injected at 1 × 10^9^ vp into QGP‐1 tumors (*n* = 6). Right, adenoviruses were injected at 2 × 10^10^ vp into QGP‐1 tumors (*n* = 6). The *P*‐value: AdSur‐infected tumors versus AdSur‐SYE‐infected tumors on day 21. (D) Transgene expression in QGP‐1 subcutaneous tumors. Frozen sections of tumors were examined by fluorescence microscopy on days 2 and 6 after injection of viruses (2 × 10^10^ vp). The sections were counterstained with DAPI. (E) Replication of adenoviral DNA in tumors. Adenoviruses were directly injected in QGP‐1 subcutaneous tumor at 2 × 10^10^ vp, and 2 and 6 days later, adenoviral DNA was analyzed by real‐time PCR (*n* = 3–4).

To examine the cytotoxic potency of AdSur‐SYE for PNET cell lines, cells were infected with viruses. An in vitro cell viability assay showed that the number of viable cells decreased day by day, and the cytotoxicity of AdSur‐SYE was somewhat higher than that of Ad‐EGFP. In PC3 cells, there was no significant difference in cell growth inhibition between AdSur‐SYE and AdSur (Fig. 5A). Five days after the viral infection, AdSur‐SYE infection resulted in a strong cell death of QGP‐1 and A99 cells, even at MOI of 100 vp/cell, whereas the effect of AdSur on PNET cells was minimal (Fig. 5B).

### In vivo antitumor effect of AdSur‐SYE

To investigate the in vivo efficacy of AdSur‐SYE, viruses were directly injected into QGP‐1 subcutaneous tumors. Infection with a low dose (1 × 10^9^ vp) of AdSur‐SYE significantly suppressed the QGP‐1 tumor growth, more than that with AdSur or AdΔE1‐AP (Fig. 5C, left panel). In general, the activity of tumor‐specific promoters is specific but less efficient than an endogenous one. Although AdSur‐SYE ablated for CAR binding and its E1 region is regulated by tumor‐specific promoter, the antitumor effect of AdSur‐SYE was compatible with that of Ad‐EGFP, which has WT E1 region and WT fiber (Fig. 5C, left panel), indicating that the SYE ligand much enhanced the infectivity and oncolytic potency for PNET. Furthermore, a high dose (2 × 10^10^ vp) of AdSur‐SYE completely eradicated QGP‐1 tumors, whereas AdSur did not suppress the QGP‐1 tumor growth (Fig. 5C, right panel). We have previously reported that no in vivo antitumor effect of AdSur‐SYE was detected in PC3 tumors, while AdSur caused an antitumor effect in PC3 tumors 14. PC3 tumors may be infected with AdSur via another unknown specific receptor, which appears in the tumor microenvironment but not in the cell culture condition, and the insertion of the SYENFSA sequence may disturb the AdSur infection due to the conformational change in a particular portion.

To examine whether the viruses replicate and spread in tumor tissues, transgene expression was compared in AdΔE1‐AP‐, AdSur‐, AdSur‐SYE‐ and Ad‐EGFP‐injected tumors on days 2 and 6 after the injection. The EGFP expression in the AdSur‐injected QGP‐1 tumors decreased on day 6 compared to that on day 2, whereas many EGFP^+^ cells were detected in the AdSur‐SYE‐injected tumors on day 2, and the number of EGFP^+^ cells was much increased and tumor cells were lytic on day 6 (Fig. 5D). EGFP^+^ cells were not detected in the adjacent skin and skeletal muscle (data not shown). Furthermore, we examined a genome copy number of adenovirus in QGP‐1 tumors on days 2 and 6 after intratumoral injection. The real‐time PCR showed that the genome copy number significantly increased in AdSur‐SYE‐injected tumors on day 6 compared to day 2, whereas the number did not increase in AdSur‐injected tumors (Fig. 5E). These results indicated that AdSur‐SYE more effectively proliferated and spread in the tumors.

All treated mice looked healthy during the course of the experiments, in the treated mice at 4 weeks. The alanine aminotransferase (ALT) level in the serum was measured 2 days post‐intratumoral administration of the virus (BioVision Incorporated, Milpitas, CA). The ALT levels were 6.62 ± 1.36, 7.90 ± 1.89, 7.55 ± 0.90, 6.07 ± 0.53, and 8.30 ± 1.67 IU/L in the mice injected with PBS, AdΔE1‐AP, AdSur, AdSur‐SYE, and Ad‐EGFP, respectively. The levels were within the normal limit in all groups. Although we previously showed that a more amount of virus genome was detected in the liver of intratumorally AdSur‐ and Ad‐EGFP‐ injected mice compared to that of AdSur‐SYE‐injected mice 14, intratumoral injection may not distribute the virus enough to cause obvious hepatic damage.

### Oncolytic activity of AdSur‐SYE in surgical specimens of human PNET

**Figure 6:**
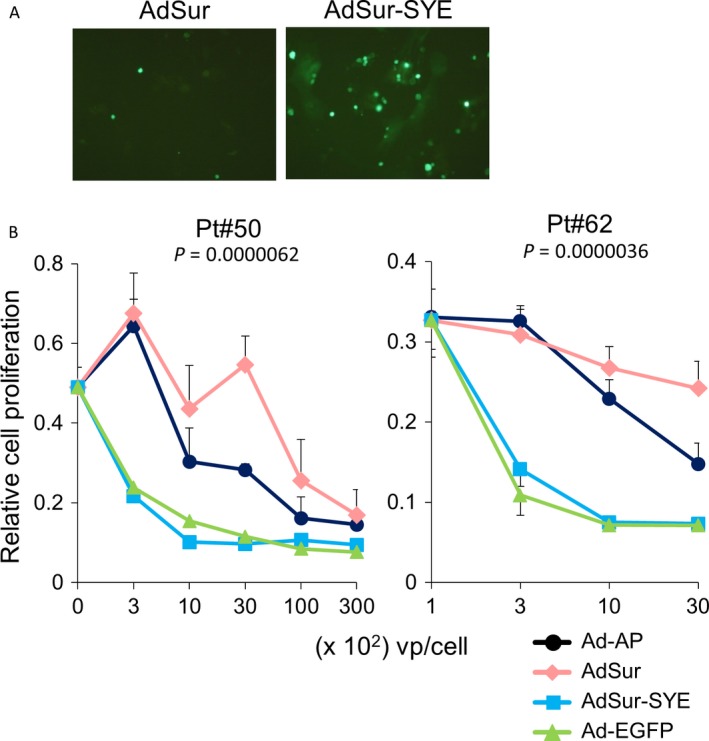
Oncolytic activity of AdSur‐SYE in human PNET tissues. Tumor cells prepared from two surgical PNET specimens (Pt#52 and Pt#62) were cocultured with MEFs and infected with adenoviruses. (A) EGFP ^+^ PNET cells. The photographs are representatives of EGFP ^+^ PNET cells (Pt#62) on day 6 after infection with AdSur‐SYE or AdSur at 1 × 10^3^ vp/cell. (B) Cytotoxic activity of adenoviruses for pancreatic cancer cells cocultured with MEFs. Six days after the infection, cell proliferation was measured by an in vitro cell viability assay. To normalize the cell proliferation by that of MEFs, differences between the OD_450_ values of PNET cells plus MEFs and those of MEFs alone are presented. The starting point was 0 vp/cell (no virus infection). The assays were carried out in four wells, and the mean ± SD was plotted. The *P*‐value: AdSur‐infected cells versus AdSur‐SYE‐infected cells at 1 × 10^3^ vp/cell.

Finally, to examine the oncolytic effect of AdSur‐SYE in human PNET tissues, single cells prepared from two patients with PNETs were cocultured with MEFs and infected with viruses. The number of EGFP^+^ cells among the AdSur‐SYE‐infected cells was larger than that among AdSur‐infected cells (Fig. 6A). An in vitro cell viability assay showed that the cancer cell proliferation was suppressed upon virus infection in a dose‐dependent manner; however, AdSur‐SYE suppressed the growth of PNET cells more effectively than AdSur (Fig. 6B). The cytotoxicity of AdSur‐SYE was equal to or greater than that of Ad‐EGFP (Fig. 6B). The results demonstrated that AdSur‐SYE showed a higher gene transduction efficiency and oncolytic activity than AdSur in human PNET as well as PDAC tissues. Although AdΔE1‐AP did not inhibit the cell growth in vitro (Fig. 5A), it showed some cytotoxicity for PNET cells (Fig. 6B). MEFs would not completely support the survival of PNET cells; the adenoviral toxicity of AdΔE1‐AP may have appeared in PNET cells on 6 days after infection.

## Discussion

We have previously reported that the pancreatic cancer‐targeting sequence SYENFSA significantly enhanced the infectivity and cytotoxic activity of an oncolytic adenovirus for pancreatic cancer cells 12, 14. In this study, we unexpectedly found that the virus with the SYE targeting ligand showed a much higher gene transduction efficiency in PNET as well as PDAC cells compared to nontargeting vector. The high infectivity of the SYE‐displaying oncolytic adenovirus led to its strong antitumor efficacy in PNET cell lines, a murine model, and clinical samples compared to that of AdSur, whereas the effect of AdSur‐SYE was almost the same as that of AdSur in other cancer and normal cells. The results indicated that AdSur‐SYE could be used in patients with PNET as well as PDAC. This study will lead to develop AdSur‐SYE for clinical trials and may change the therapeutic strategy of PNET in the foreseeable future.

The high gene transduction efficacy of the SYE‐displaying adenovirus for both PDAC and PNET indicates that the tumors may share the same receptors for the SYE ligand, even though their histological origins are different. The receptor molecules may be associated with a common pathway of tumorigenesis of PDAC and PNET. The identification of the receptors will be important for understanding of molecular characteristics of target cells and can be used for the diagnosis such as detection of the disease relapse and to develop a new treatment strategy. Currently, we conduct research to identify receptors for the SYE ligand in various cancer cells using a microarray technology.

In general, surgical resection is used as a first‐line treatment of the primary PNET. Complete resection leads to a 5‐year survival rate of 90% to 100% in patients with PNETs. Incomplete resection reduces the rate to 20–75%, and occurring hepatic metastasis further reduces the rate to 10–50% 16, 17. PNETs are pathologically heterogenous and categorized into three groups (neuroendocrine tumors, NET G1 and NET G2, and neuroendocrine carcinoma, NEC). Conventional chemotherapy includes regimens based on etoposide, platinum agents, anthracyclines, streptozotocin, 5‐fluorouracil‐based agents, and somatostatin analogs 15. Recently, the targeting agents sunitinib malate and everolimus received the U.S. FDA approval for the treatment of PNET in patients with advanced NET G1/G2 15. However, since effective therapeutic options are still lacking for patients with advanced PNET and the clinical outcome of NEC is dismal, these patients require a new type of therapy. For locally advanced cases, it is important to develop strategies to strongly control local lesions and prevent distant metastases. In pancreatic cancer, regional therapy is particularly relevant because locally advanced cases can be accessible by ultrasound‐ or computed tomography‐guided percutaneous injection or endoscopic ultrasound‐guided injection. A strong control of local lesions by the injection of targeting oncolytic adenoviruses may prevent the occurrence of distant metastasis, leading to the improvement of patient survival.

To regulate the replication of the adenovirus, we used the survivin promoter in the AdSur‐SYE construct. Survivin is one of antiapoptotic proteins involved in mitotic regulation during embryonic and fetal development, but its expression is generally undetectable in somatic cells 20. Thus, survivin is a broad‐spectrum molecular target for cancer therapy. In nude mouse xenograft experiments involving liver and gallbladder cancers, survivin promoter‐regulated oncolytic adenoviruses replicated in and lysed cancer cells in a targeted manner 20. In PNET, clinical studies have indicated a correlation between high survivin expression levels and a poor survival prognosis; patients with 5%, 5–50%, and > 50% positive nuclei had a median survival of 225, 101, and 47 months, respectively 21, 22. This implies that survivin‐driven viral treatment can work better for a patient with a more malignant tumor since the virus could replicate effectively in high survivin‐expressing cells.

One of the most important issues for achieving success in clinical trials is the selection of patients with tumor tissues that are suitable for this targeting strategy. Selection of patients based on a high viral infectivity and high survivin expression in biopsy samples might be feasible in a clinical setting 7. If a list of several PNET‐targeting, cancer‐specific promoter‐driven viruses is generated, the most suitable virus could be selected for each patient based on the viral infectivity and promoter activity in biopsy samples and patient‐derived xenografts, ultimately leading to personalized oncolytic therapy.

In conclusion, AdSur‐SYE exerted a high gene transduction efficiency and strong antitumor efficacy for PNET as well as for PDAC. Thus, AdSur‐SYE is a promising next‐generation therapy for advanced PNET.

## Conflict of Interest

The authors have no conflict of interest to declare.

## Supporting information


**Figure S1.** Frequency of EGFP^+^ and Chromogranin A^+^ cell population in PNET tumors.

**Table S1.** Clinicopathological findings of clinical samples.
