# Supplementary figures and images for: Strong antitumor efficacy of a pancreatic tumor‐targeting oncolytic adenovirus for neuroendocrine tumors
Source: Cancer Med. 2017 Sep 21;6(10):2385–97. doi: 10.1002/cam4.1185 (PMC5633550; doi:10.1002/cam4.1185)

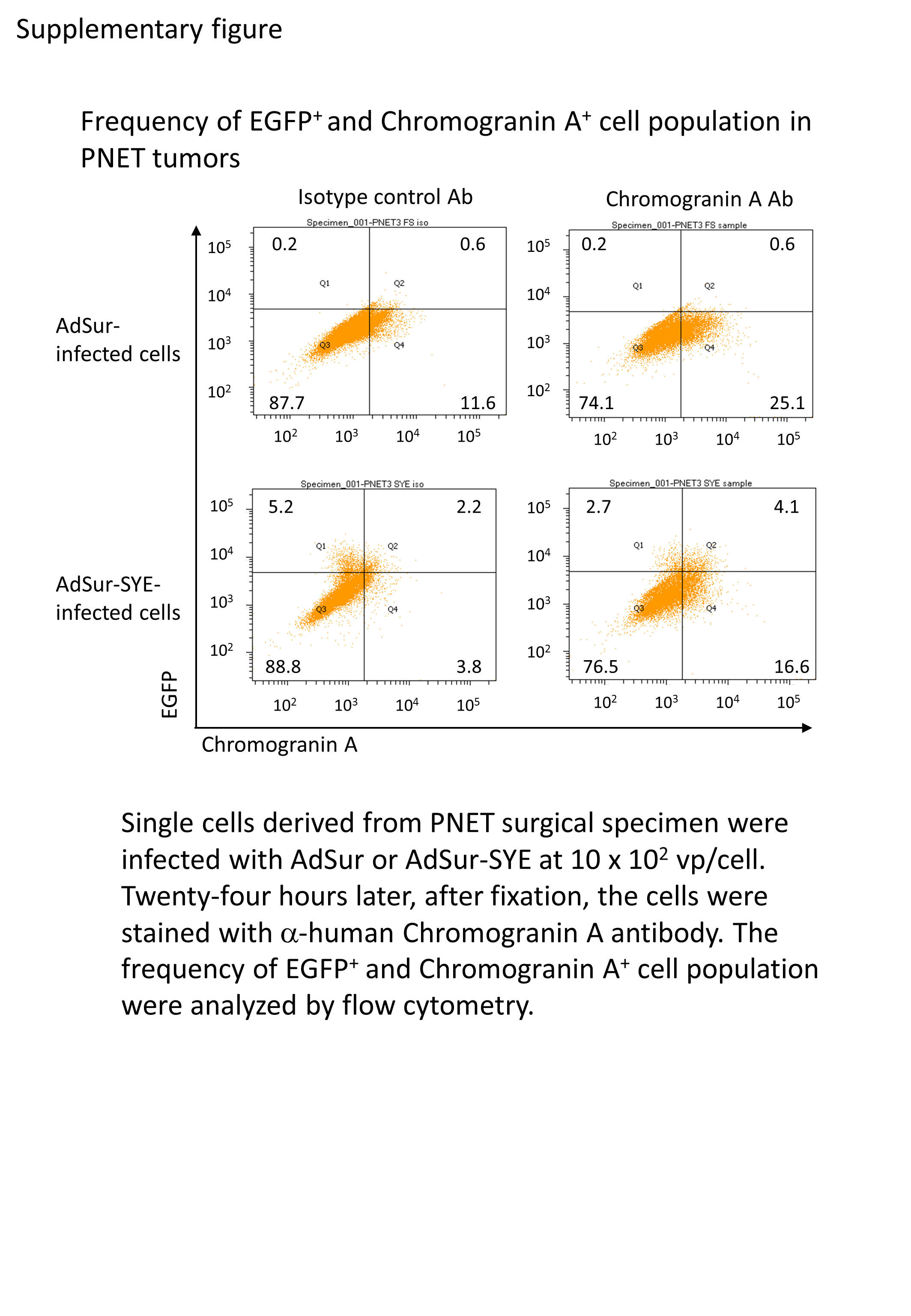

Supplement: Supplementary file 1 — Figure S1. Frequency of EGFP+ and Chromogranin A+ cell population in PNET tumors. [file CAM4-6-2385-s001.TIF]

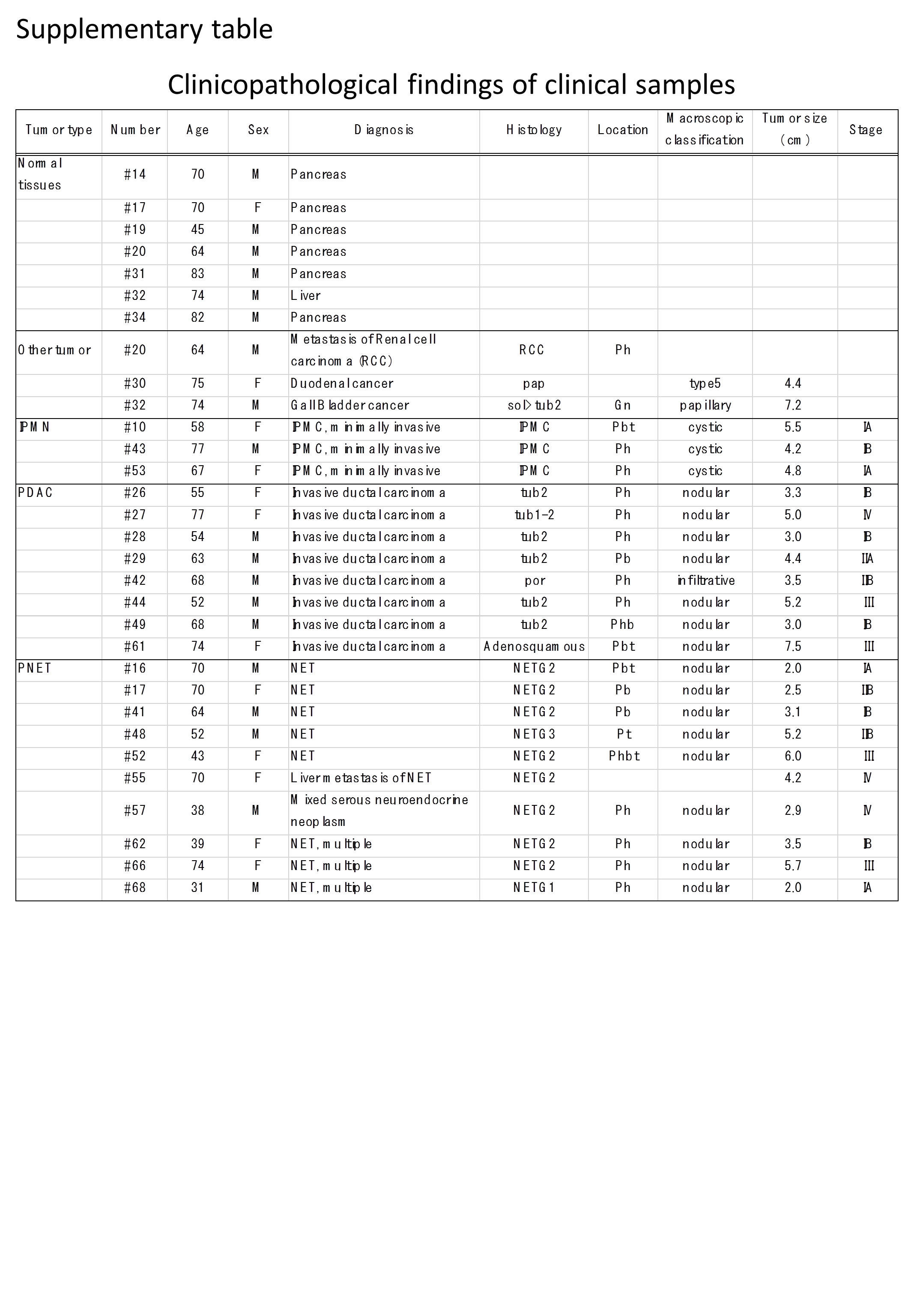

Supplement: Supplementary file 2 — Table S1. Clinicopathological findings of clinical samples. [file CAM4-6-2385-s002.TIF]
